# Connectivity properties in the prefrontal cortex during working memory: a near-infrared spectroscopy study

**DOI:** 10.1117/1.JBO.24.5.051410

**Published:** 2019-03-21

**Authors:** Jinyan Sun, Fang Liu, Haixian Wang, Anping Yang, Chenyang Gao, Zhicong Li, Xiangning Li

**Affiliations:** a Foshan University, School of Medical Engineering, Department of Biomedical Engineering, Foshan, China; b Foshan University, School of Mathematics and Big Data, Foshan, China; c Institute of Biomedical Engineering, Chinese Academy of Medical Sciences & Peking Union Medical College, Tianjin, China; d Guangdong Medical University, Department of Biomedical Engineering, Dongguan, China; e Huazhong University of Science and Technology, Britton Chance Center for Biomedical Photonics, Wuhan National Laboratory for Optoelectronics, Wuhan, China; f HUST-Suzhou Institute for Brainsmatics, Suzhou, China

**Keywords:** near-infrared spectroscopy, working memory, brain connectivity, prefrontal cortex, n-back

## Abstract

Working memory (WM) plays a crucial role in human brain functions. The application of brain connectivity analysis helps to understand the brain network properties in WM. Combination of functional and effective connectivity can provide new insights for exploring network attributes. Nevertheless, few studies have combined these two modes in WM. Near-infrared spectroscopy was used to investigate the connectivity properties in the prefrontal cortex (PFC) during n-back (0-back and 2-back) tasks by combining functional and effective connectivity analysis. Our results demonstrated that the PFC network showed small-world properties in both WM tasks. The characteristic path length was significantly longer in the 2-back task than in the 0-back task, while there was no obvious difference in the clustering coefficient between two tasks. Regarding the effective connectivity, the Granger causality (GC) was higher for right PFC→left PFC than for left PFC→right PFC in the 2-back task. Compared with the 0-back task, GC of right PFC→left PFC was higher in the 2-back task. Our findings show that, along with memory load increase, long range connections in PFC are enhanced and this enhancement might be associated with the stronger information flow from right PFC to left PFC.

## 1 Introduction

Working memory (WM) has been defined as a short-time memory system that is responsible for temporarily maintaining and manipulating information. During WM, the frequent renewal and rapid extraction of information occurs. WM plays a key role for complex cognitive functions of the human brain.^1^^–^^3^ n-Back is a commonly used task for the study of WM. Using the n-back task, several brain regions have been identified as robustly activated brain areas for WM, including dorsolateral and ventrolateral prefrontal cortex (PFC), frontal pole, parietal cortex, dorsal cingulate/medial premotor cortex, etc.^4^

Using the n-back task, many previous studies emphasized investigating the activation of related brain regions during different types of WM tasks (e.g., verbal, spatial, or object) and the relationship between the activation and memory load.^4^^–^^6^ Since the brain generally carries out brain functions in the form of a network,^7^ brain connectivity is considered as an important tool for understanding the human brain function. Brain connectivity provides unique information complementing the brain activation.^8^ At present, there’s increasing research on the investigation of brain connectivity in WM and the influence of memory load on brain connectivity.^2^^,^^9^ For instance, Sala-Llonch et al.^2^ found a strong correlation between default mode network and WM network in the difficult 3-back task, which could predict behavior.

Small-worldness analysis is another commonly used method for the study of brain connectivity during WM. Small-worldness analysis, based on complex network analysis which is an extension of graph theory, is an important tool for analyzing brain functional connectivity. Using this method, several issues, such as network connectivity and efficiency, can be considered in an integrated manner. Clustering coefficient (C) and characteristic path length (L) are commonly used network metrics that represent small-worldness. C is defined as the number of connections that exist between the nearest neighbors of a node as a proportion of the maximum number of possible connections. It reflects the clustering ability of nodes in the network. L is the average distance between two points in the network, which reflects the global connectivity characteristic of the network. Compared with random network, the absolute characteristic path length is almost the same and the absolute clustering coefficient is higher in small-world network, that is $\lambda =L^{\mathrm{real}}/L^{\mathrm{rand}}\approx 1$,$\gamma =C^{\mathrm{real}}/C^{\mathrm{rand}}>1$. It has been implicated that the brain functional network has small-world properties in WM and these small-world properties could be affected by various factors such as memory load, diseases, and age.^10^^–^^12^

In addition to functional connectivity, effective connectivity is also an important analysis method for studying brain connectivity. Effective connectivity is a directed connectivity that can be used to investigate the influence of one neuronal system on another one, either at a synaptic or population level.^13^ Moreover, effective connectivity has been used for the assessment of brain connectivity in n-back tasks in emerging lines of studies. Using the n-back task combined with dynamic causal modeling, Dima et al. demonstrated that the effective connectivity between brain regions involved in WM was affected by memory load. In moderate (2-back) and high difficult (3-back) tasks, the information flowed from posterior parietal lobe to anterior frontal lobe, and evident right-hemisphere dominance was detected. However, the right-hemisphere dominance was not evident in the less difficult (1-back) task.^14^ Using immediate and delayed memory tasks, Ma et al. found an enhanced connection from left posterior parietal cortex to left inferior frontal gyrus with increased memory load.^9^ Besides, the effective connectivity between brain regions involved in WM could also be affected by other factors, such as diseases.^15^

These studies collectively emphasized the importance of brain connectivity analyses in studying WM. Functional connectivity is an observable phenomenon that can be quantified by statistical dependencies, such as correlations. However, effective connectivity corresponds to the parameter of a model that tries to explain observed dependencies.^16^ The combined application of these two methods may provide new insights for exploring the brain connectivity in WM. Nevertheless, until recently, few studies analyzed the small-world property and effective connectivity in brain network simultaneously during n-back tasks.

In this study, the connectivity properties in the PFC which is an important brain region involved in WM was investigated with near-infrared spectroscopy (NIRS). NIRS is a developing and promising noninvasive optical functional neuroimaging method and can measure the concentration changes of oxyhemoglobin ($\Delta [{\mathrm{HbO}}_{2}]$) and deoxyhemoglobin (Δ[Hb]) to reflect relative regional brain activity. NIRS can provide high temporal resolution and reasonable spatial resolution. Along with the development of methods for processing NIRS data,^17^^,^^18^ NIRS has been wildly used in cognitive neuroscience due to its flexibility and high ecological validity.^19^^,^^20^ In recent years, with the development of multichannel NIRS systems, accumulative evidence indicates that NIRS is also an efficient tool for assessment of brain connectivity.^21^^–^^26^

So far, previous NIRS studies investigated WM with n-back tasks mainly with brain activation analysis,^5^^,^^27^^,^^28^ but much less abundantly with brain connectivity analysis.^29^ The PFC connectivity properties in WM have not been fully explored. In this study, NIRS was used to analyze the small-world property and directional connectivity in PFC simultaneously during n-back tasks to explore the characteristics of brain connectivity in PFC during WM.

## 2 Method

### 2.1 Subjects

A total of 15 paid subjects were recruited in this study. Among these subjects, seven were female. They ranged from 20- to 26-years old, with a mean age of 22.9 years. All participants were right handed and had normal or corrected-to-normal vision. All were healthy and had no physiological or psychological disorders. The experimental procedures were explained in detail to each participant before test. Prior to the test, participants underwent a preliminary experiment to ensure that they were familiar with the experimental procedures. Each participant signed an informed consent before the experiment. This experiment was approved by the Human Subjects Institutional Review Board at Huazhong University of Science and Technology. The NIRS data of two male subjects were excluded for analysis because of relatively large noise.

### 2.2 Materials and Procedures

**Fig. 1:**
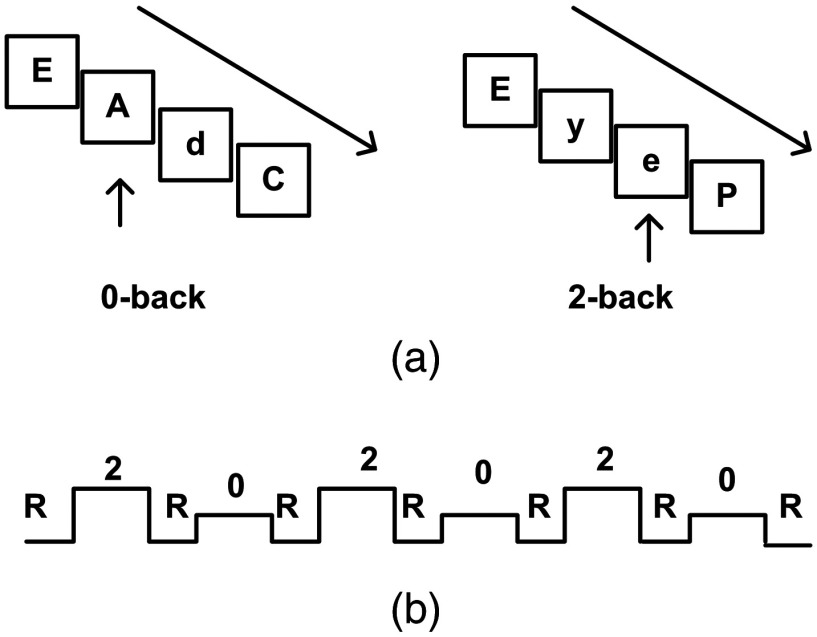
Experimental design of the n-back task. (a) The stimuli sequence. Arrows indicate the targets in 0-back (upper left) and 2-back (upper right) tasks. (b) The task sequence. R, 1-min rest period before and after each task block; 2, 90-s 2-back task block; 0, 90-s 0-back task block.

The n-back (0-back and 2-back) task with block design was used. In each task block, participants were given 30 letters. Each letter was presented for 500 ms. After a 2.5-s interval (a blank screen), the next letter was presented. During the 0-back task, participants were asked to press the key when the letter “a” or “A” presented. During the 2-back task, participants were asked to press the key when a currently displayed letter matched the two preceding letter (regardless of case). In each task block, 10 targets were set. There were six task blocks in the experiment with 2-back and 0-back tasks alternatively presented. The task sequence was shown in Fig. 1. Participants were allowed to rest for 60 s before and after each task block.

### 2.3 NIRS Recording

**Fig. 2:**
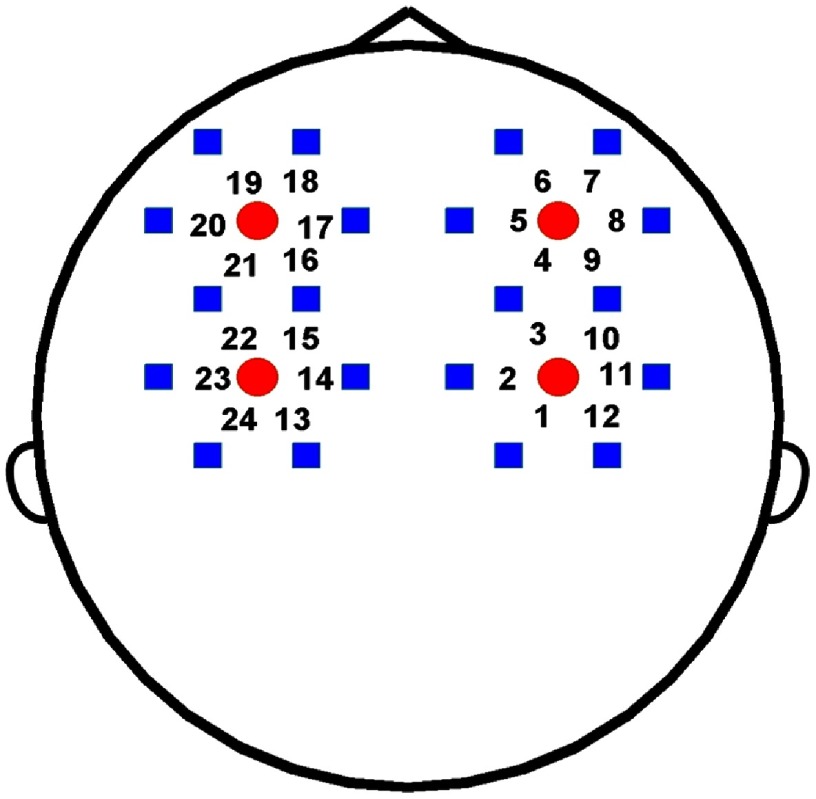
Schematic of NIRS measurement channel locations on the head. The red circle indicates the NIRS light source and the blue rectangle indicates the detector. The number (1 to 24) denotes the NIRS detective channel (midpoint of the source-detector pair).

The NIRS data were acquired by the continuous-wave functional NIRS system developed by Britton Chance Center for Biomedical Photonics independently.^30^ The system uses two wavelengths (785 and 850 nm) to determine the concentration changes of ${\mathrm{HbO}}_{2}$ and Hb based on the modified Beer–Lambert law. The NIRS probe contains four light sources and 20 light detectors, forming 24 detective channels (Fig. 2). The distance between the light source and the detector is 3 cm. The sampling frequency is 100 Hz. The NIRS probe was placed according to the position of two EEG electrodes, F3 and F4. Specifically, the NIRS channel 4 (Ch 4) was over the F4 electrode, and Ch 16 was over the F3 electrode. The line through the two light sources in the same lateral hemisphere was parallel to the midline (i.e., the arc from the nasion to the inion).

### 2.4 NIRS Data Processing

**Fig. 3:**
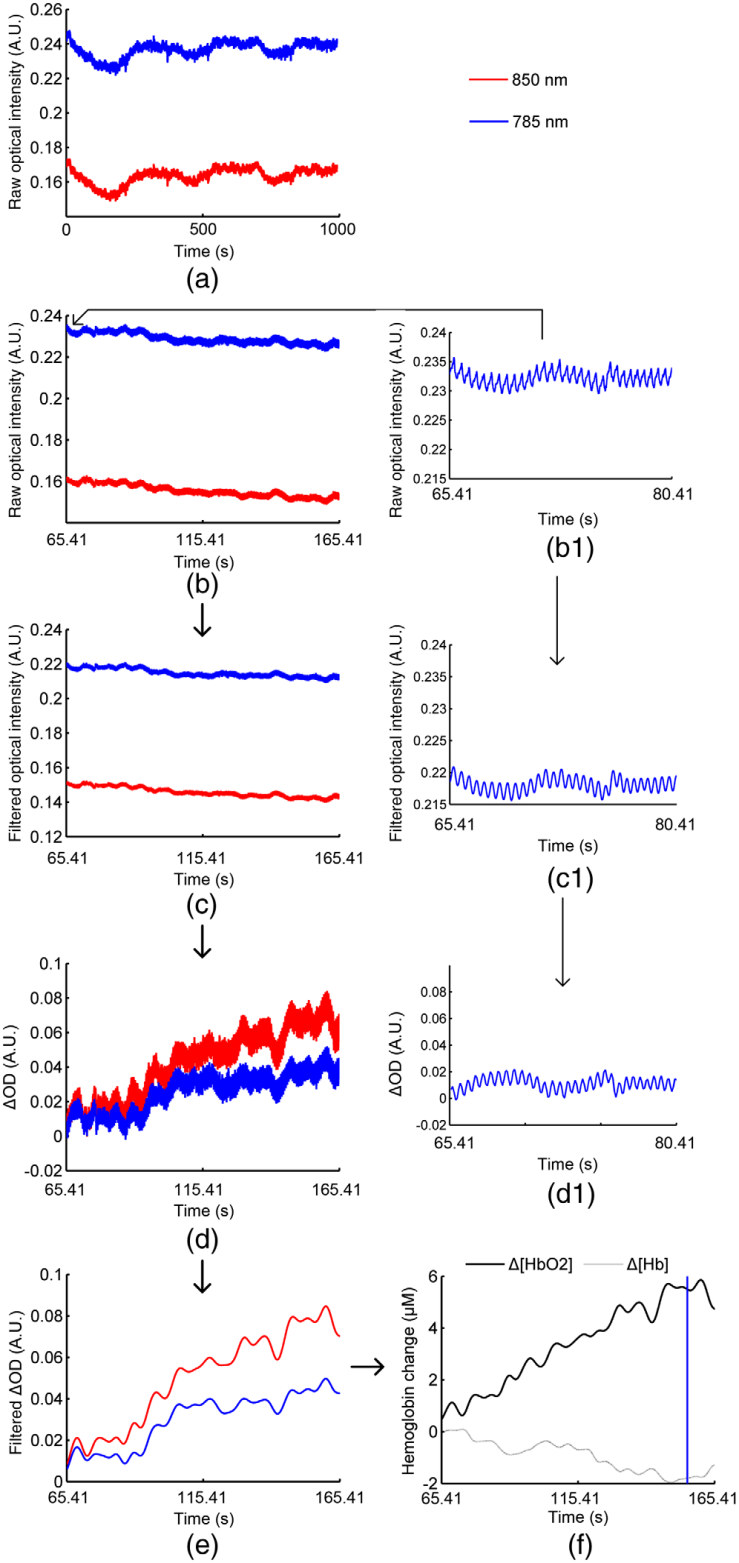
Illustration of NIRS data processing. (a) The raw optical intensity during the entire experiment detected from 850 nm (red) and 785 nm (blue) measurement at one typical channel for one subject. Thereinto, the raw optical data from the first task block beginning to 10 s after the first task block end are shown in (b). Data in (b) were low-pass filtered at (c) 3 Hz, downsampled to 10 Hz, and (d) converted to change in ΔOD. (e) ΔOD was band-pass filtered (0.01 to 0.2 Hz) and (f) converted to hemodynamic parameters. The blue line in (f) shows the first task block end. (b1), (c1), and (d1) give the enlarged view for data of 785 nm in (b), (c), and (d) during a period of 15 s.

To eliminate high-frequency noise generated by equipment, the raw light intensity data were initially low-pass filtered at 3 Hz (least-squares FIR filter with zero-phase distortion; order: 50). After that, the frequency was declined to 10 Hz. After transforming the light intensity into optical density changes (ΔOD), ΔOD was band-pass filtered (0.01 to 0.2 Hz) to eliminate the low-frequency shift and physiological noise, and then converted into hemodynamic parameters ($\Delta [{\mathrm{HbO}}_{2}]$ and Δ[Hb]) using the differential pathlength factor (DPF) method. The DPF values at 785 and 850 nm were set as 6.0 and 5.2, respectively.^31^^,^^32^ The NIRS data processing is shown in Fig. 3. Since the amplitude and the signal-to-noise ratio is higher in $\Delta [{\mathrm{HbO}}_{2}]$ than Δ[Hb],^33^ we here only analyzed brain activation and connectivity based on ${\mathrm{HbO}}_{2}$ response. The mean values of ${\mathrm{HbO}}_{2}$ for the baseline (the last 20-s rest before each task) and the task (the entire 90-s task block) were computed for each channel, task condition, and subject. The hemodynamic response for each task was indicated by the difference in the mean values of the ${\mathrm{HbO}}_{2}$ signal between the task and the baseline. Then, for each subject, the hemodynamic parameters were block averaged for each task condition separately. T tests were used for statistical analysis of hemodynamic response.

### 2.5 Small-World Properties

In order to analyze the small-world properties, the correlation coefficients between all pairwise combinations of NIRS channels were calculated, yielding a 24*24 matrix. In order to improve the normal distribution pattern, a Fisher’s r-to-z transformation was performed on correlation coefficients matrix. Threshold value T was set and the Z value matrix is converted into binary network. When T was set as 0.6, the first 40% absolute values of Z were defined as 1, and the others were defined as 0. 1 represents connectivity between two channels, that is, there is an edge between two channels. Such threshold setting method ensures the same number of edges in different networks when comparing two tasks. Using Matlab Brain Connectivity Toolbox (BCT),^34^ C and L were calculated when T value was between 0.3 and 0.9 (step size = 0.01). We first clarified whether or not small-world property existed in the brain network of PFC in WM tasks. After that, the 2-back task was compared with the 0-back task.

### 2.6 Effective Connectivity

Effective connectivity was examined using the Granger causality (GC) method. GC is based on the general concept that a variable X1 Granger causes X2 if predictions of the value of X2 based on the past values of X1 are better than predictions of X2 based only on its own past values. In the analysis of brain connectivity, GC values of structure 1→structure 2 higher than structure 2→structure 1 indicate a stronger functional influence from structure 1 on structure 2. GC method has been shown to be suitable for studying directional connectivity on the cerebral blood oxygen responses.^22^^,^^24^

Effective connectivity analysis was conducted by referring to previous published NIRS studies.^22^^,^^24^ Briefly, the GC values of homologous channel pairs (e.g., Ch 1 and Ch 13) in left and right PFC were calculated by using GC Matlab toolbox.^13^ The calculation processes for GC included data preprocessing, data stationary check, model validity, and consistency verification. GC calculation is based on the multivariate autoregressive modeling (AR) of the signal. The order of AR defined the maximum number of lagged observations included in the model and was chosen based on the Bayesian information criterion. For an AR order of 10, the corresponding timelag was 1 s. Different timelags (1, 2, 3, 4, and 5 s) were used to confirm that the results were robust. For each subject, 12 left PFC→right PFC GC values were generated, and averaged to evaluate the overall influence of left PFC on right PFC at each timelag, and vice versa. The repeated-measures ANOVA was carried out for GC values with task (0-back, 2-back) × laterality (left PFC→right PFC, right PFC→left PFC) as within-subject factors.

## 3 Results

### 3.1 Behavioral Results

Compared to that in the 0-back task, the response time was extended, and the accuracy was reduced in the 2-back task (p<0.05).

### 3.2 Brain Activation Results

**Fig. 4:**
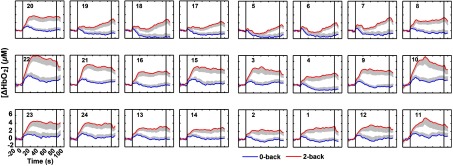
The grand average time courses of ${\mathrm{HbO}}_{2}$ responses in the 0-back (blue) and 2-back (red) tasks at all channels. The number in each subgraph indicates the NIRS channel number. The black lines at 0 and 90 s show the start and end of the task blocks. The labels and scales of the axes are the same for all subgraphs. The gray region indicates standard error of the mean (SEM).

Figure 4 shows the grand average time courses of ${\mathrm{HbO}}_{2}$ responses to 0-back and 2-back tasks at all channels. An evident activation of HbO_2_ in PFC was observed during the 2-back task. Except for Ch 5/6/17/18/19, significant positive activation was detected in all other channels in the 2-back task (p<0.05, FDR correction). In contrast, in the 0-back task, the concentration of ${\mathrm{HbO}}_{2}$ was increased during the initial phase of task and gradually decreased after activation (Fig. 4). For the 0-back task, there was significant positive activation in Ch 21 (p<0.05), and marginally significant positive activation in Ch 10/22 (p<0.1). Compared with that in the 0-back task, $\Delta [{\mathrm{HbO}}_{2}]$ was significantly larger in all channels in the 2-back task (p<0.05, FDR correction).

In the 2-back task, the mean activation of the left PFC channels was slightly stronger than right PFC homologous channels except for Ch 19 and Ch 23, while significant enhancement of activation was only detected in Ch 17 when compared to Ch 5 (p<0.05). In the 0-back task, significant enhancement of activation was only detected in Ch 21 when compared to Ch 9 (p<0.05). These results indicate that there is no obvious laterality effect for the PFC activation undergoing WM task.

### 3.3 Small-World Properties Results

**Fig. 5:**
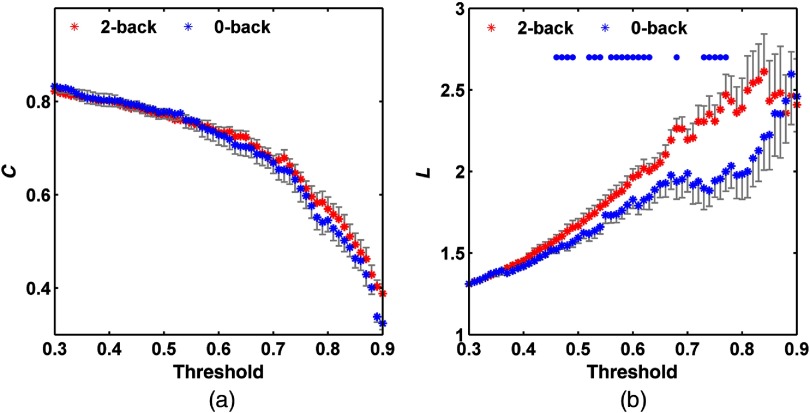
(a) Mean clustering coefficient C and (b) characteristic path length L for 0-back and 2-back tasks as a function of threshold. Error bars indicate SEM. Blue circles in (b) indicate significant difference in L values between two tasks (t test, p<0.05).

Figure 5 shows the variation tendency of C and L values with various threshold T (0.3 to 0.9). When the threshold value is small, the corresponding binary network is mostly connected with edges between most channel pairs. With the increase of threshold value, C value is gradually decreased with the loss of edges. Besides, the increase of threshold value results in increase in the average path length due to the loss of edges. Therefore, L value increases with threshold value.

Average γ ($\gamma =C^{\mathrm{real}}/C^{\mathrm{rand}}$) and λ ($\lambda =L^{\mathrm{real}}/L^{\mathrm{rand}}$) values under various thresholds are listed in Table 1. It should be noted that, when T value was between 0.8 and 0.9, the λ value was lower than 1, which was not provided in Table 1. Table 1 reveals that when T value is between 0.5 and 0.8, γ>1 and λ≈1 in both 2-back and 0-back tasks, in which case the PFC network shows small-world properties.

**Table 1:** Average γ and λ values under various threshold values.

Task	0.3 to 0.4	0.41 to 0.5	0.51 to 0.6	0.61 to 0.7	0.71 to 0.8
2-back	γ=1.09	γ=1.16	γ=1.42	γ=1.68	γ=2.14
	λ=1.00	λ=1.03	λ=1.16	λ=1.23	λ=1.22
0-back	γ=1.06	γ=1.10	γ=1.27	γ=1.48	γ=1.79
	λ=1.01	λ=1.02	λ=1.09	λ=1.12	λ=1.03

There was no significant difference in C values between two tasks as determined by t tests. However, the L value in the 2-back task was larger than that in the 0-back task (Fig. 5).

### 3.4 Effective Connectivity Results

**Fig. 6:**
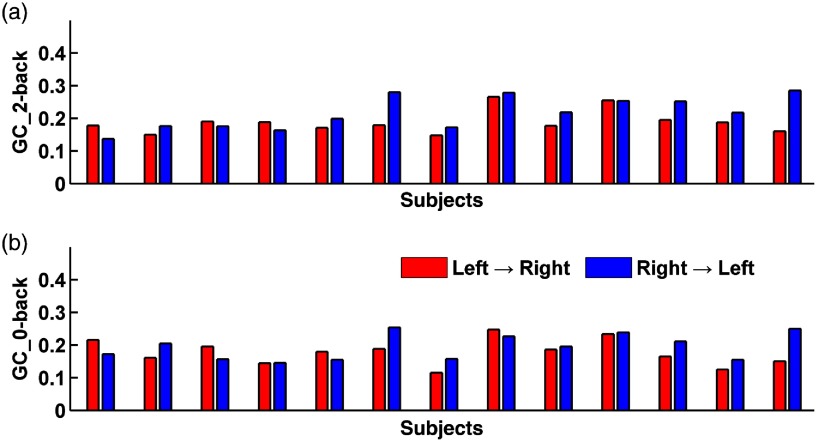
The average GC values derived from all participants undergoing (a) 2-back and (b) 0-back tasks. Timelag = 4 s. Red, left PFC→right PFC; blue, right PFC→left PFC.

Figure 6 shows the GC values derived from all participants undergoing 2-back and 0-back tasks with the timelag of 4 s. Repeated-measures ANOVA was carried out for GC values under each timelag. The ANOVA revealed a significant task effect [F(1,12)=5.533, p=0.037], and a marginally significant laterality effect [F(1,12)=3.5, p=0.086] when timelag was 4 s. T test further showed that the GC value of right PFC→left PFC was higher than that of left PFC→right PFC in the 2-back task (p=0.05). Moreover, the GC value of right PFC→left PFC was higher in the 2-back task than that in the 0-back task (p=0.016).

When timelag was 3 s, a marginally significant task effect was detected [F(1,12)=4.089, p=0.066]. Further T tests yielded similar results as that in timelag of 4 s. The GC value of right PFC→left PFC was higher than that of left PFC→right PFC in the 2-back task (p=0.05). Moreover, the GC value of right PFC→left PFC was higher in the 2-back task than that in the 0-back task (p=0.027).

## 4 Discussion

In this study, we investigated the small-world properties and effective connectivity in the PFC during n-back tasks. Our results demonstrated that the measured PFC area was significantly activated during the 2-back task. The PFC network showed small-world properties in WM tasks. The characteristic path length was significantly increased in the 2-back task than in the 0-back task, while there was no obvious difference in clustering coefficient between two different tasks. Regarding the effective connectivity in the 2-back task, the GC value was higher for right PFC→left PFC than for left PFC→right PFC. In addition, compared with that in the 0-back task, the GC value of right PFC→left PFC was higher in the 2-back task.

There was strong hemodynamic activation in the 2-back task. While, most channels show ${\mathrm{HbO}}_{2}$ concentration increase after the beginning of the 0-back task. As the 0-back task went on, the ${\mathrm{HbO}}_{2}$ concentration decreased due to the low cognitive load. These activation results are consistent with previous n-back studies with NIRS^35^^,^^36^ and demonstrate WM-related frontal activation.

PFC is an essential brain region participating in various cognitive functions including WM. Its small-world properties have been confirmed during resting state and several cognitive functions.^21^^,^^37^^,^^38^ In line with these findings, we here reported that the PFC network showed the small-world properties during n-back tasks. The small-world properties are attributed by the locally clustered connections and loosen long range connections.^8^ Compared with the 0-back task, the 2-back task had larger memory load. With increased memory load, there was no significant change in C value, indicating that the locally clustered connections in PFC was not altered. Nevertheless, the L value was increased with memory load, suggesting the enhanced long range connections and complexity in PFC. These findings were consistent with previous reports. For instance, functional magnetic resonance imaging (fMRI) was utilized for studying the global small-world properties by Ginestet and Simmons. Their results revealed that, within certain network cost range, the global efficiency which was inversely related to L value, was higher in the 0-back task than in the 2-back task.^12^ He et al. used fMRI for studying the Sternberg item recognition performance. They found that the average L value was elevated with increased memory load as the average L value in the three-item memory task was higher than that in the one-item memory task.^11^ These data imply that increased memory load not only results in the enhanced activation of the left and right PFC, but also increased endogenous long range connection and network complexity, which ensures the efficient information delivery and mission completion.

Effective connectivity analysis showed that there was no significant difference in the GC values between right PFC→left PFC and left PFC→right PFC in the 0-back task. However, the GC value of right PFC→left PFC was significantly higher than that of left PFC→right PFC in the 2-back task. These data indicate a stronger information flow from right PFC to left PFC with increased WM load, in spite of a slight stronger activation in left PFC than in right PFC in the 2-back task. The results of effective connectivity analysis imply the existence of right-hemisphere dominance as the influences of right PFC over left PFC appears to be stronger than left PFC over right PFC. These findings were consistent with Dima’s study. Dima et al. showed that right-hemisphere dominance existed in WM network with increased memory load. The effective connectivity from right parietal lobe toward right dorsolateral PFC (DLPFC) accounts for the dominant position. Moreover, the effective connectivity from right DLPFC toward left DLPFC is also stronger than that from left DLPFC toward right DLPFC.^14^ The effective connectivity results further emphasize the informative value of brain connectivity being not identical to brain activation.

Functional connectivity is an observable phenomenon that can be quantified by statistical dependencies, such as correlations. However, effective connectivity corresponds to a model parameter that tries to explain observed dependencies.^16^ In this study, the combined analysis of the functional and effective connectivity reveals that the enhanced long range connections in PFC with increasing memory load might be partially associated with the stronger right-to-left information flow in PFC undergoing WM tasks. Previous NIRS studies investigated WM mainly with brain activation analysis,^5^^,^^27^^,^^28^ but much less abundantly with brain connectivity analysis.^29^ This study improves our understanding about connectivity properties in the PFC during WM and is an useful application of NIRS in WM with n-back tasks.

PFC is an important brain region involved in WM. The abnormal activation or network attributes in PFC has been implicated in various disorders, such as Schizophrenia.^39^ In this study, the network attributes of PFC during WM were investigated. Our study has a positive guiding significance in understanding the function of PFC in WM or in formulating interventions for patients. However, our current study also has some limitations. Here, only 0-back and 2-back tasks were used. The small-world attributes and effective connectivity results in PFC should be confirmed in more memory load tasks (e.g., 1-back and 3-back tasks). In addition, connectivity differences between task conditions were the focus of this study. And the evaluation of differences would reduce the influence of task-unrelated signal components.^40^ The influence of superficial signal would be reduced during evaluation of differences between 0-back and 2-back tasks, and the differences are caused by neuronal activity.^23^^,^^40^ Based on this, the superficial signal in NIRS data was not particularly processed. In further studies, we will try to use adaptive filtering by adding reference channels with a short source-detector distance to reduce the superficial signal.^41^

In this study, we investigated the brain connectivity properties in PFC undergoing WM tasks with NIRS. Our findings reveal that small-world properties exist in the PFC network undergoing WM tasks. The long range connections in PFC enhance with increasing memory load, which is possibly associated with stronger right-to-left PFC information flow. Our study demonstrates that combined analysis of functional and effective connectivity provides unique information for better understanding the brain network attributes of cognitive function. This study helps to promote the application of NIRS in cognitive and clinical neuroscience.
